# Etiology of bacterial pneumonia and multi-drug resistance pattern among pneumonia suspected patients in Ethiopia: a systematic review and meta-analysis

**DOI:** 10.1186/s12890-024-03000-1

**Published:** 2024-04-16

**Authors:** Mihret Tilahun, Melaku Ashagrie Belete, Alemu Gedefie, Habtu Debash, Ermiyas Alemayehu, Daniel Gebretsadik, Hussein Ebrahim, Ousman Mohammed

**Affiliations:** Department of Medical Laboratory Sciences, College of Medicine and Health Sciences, Wollo University, Dessie, Ethiopia

**Keywords:** Prevalence, Bacterial pneumonia, Multidrug resistance, Systematic review and meta-analysis, Ethiopia

## Abstract

**Background:**

Bacterial pneumonia can affect all age groups, but people with weakened immune systems, young children, and the elderly are at a higher risk. *Streptococcus pneumoniae*, *Klebsiella pneumoniae*, *Haemophilus influenzae*, and *Pseudomonas aeruginosa* are the most common causative agents of pneumonia, and they have developed high MDR in recent decades in Ethiopia. This systematic review and meta-analysis aimed to determine the pooled prevalence of bacterial pneumonia and multidrug resistance in Ethiopia.

**Methods:**

The articles were searched extensively in the electronic databases and grey literature using entry terms or phrases. Studies meeting the eligibility criteria were extracted in MS Excel and exported for statistical analysis into STATA version 14 software. The pooled prevalence of bacterial pneumonia and multidrug resistance were calculated using a random-effects model. Heterogeneity was assessed by using the I^2^ value. Publication bias was assessed using a funnel plot and Egger’s test. A sensitivity analysis was done to assess the impact of a single study on the pooled effect size.

**Result:**

Of the 651 studies identified, 87 were eligible for qualitative analysis, of which 11 were included in the meta-analysis consisting of 1154 isolates. The individual studies reported prevalence of bacterial pneumonia ranging from 6.19 to 46.3%. In this systematic review and metanalysis, the pooled prevalence of bacterial pneumonia in Ethiopia was 37.17% (95% CI 25.72–46.62), with substantial heterogeneity (I^2^ = 98.4%, *p* < 0.001) across the studies. The pooled prevalence of multidrug resistance in bacteria isolated from patients with pneumonia in Ethiopia was 67.73% (95% CI: 57.05–78.40). The most commonly isolated bacteria was *Klebsiella pneumoniae*, with pooled prevalence of 21.97% (95% CI 16.11–27.83), followed by *Streptococcus pneumoniae*, with pooled prevalence of 17.02% (95% CI 9.19–24.86), respectively.

**Conclusion:**

The pooled prevalence of bacterial isolates from bacterial pneumonia and their multidrug resistance were high among Ethiopian population. The initial empirical treatment of these patients remains challenging because of the strikingly high prevalence of antimicrobial resistance.

**Supplementary Information:**

The online version contains supplementary material available at 10.1186/s12890-024-03000-1.

## Introduction

Pneumonia is an infection-induced inflammation of the lung tissue due to infectious caused by bacteria and other agents [1]. There is a very wide variety of pneumonia-responsible pathogens with the largest agents are bacteria [2] and resulting in approximately 7 million deaths annually [3].The most common causative agents are *Streptococcus pneumoniae* (*S. pneumoniae)*, *Haemophilus influenzae* (*H. influenzae)*, *Klebsiella pneumoniae (K. pneumoniae)*, *Pseudomonas aeruginosa* (*P*. *aeruginosa), Escherichia coli (E. coli) and Staphylococcus aureus (S. aureus)* [4]. In Spain, S. *pneumoniae* was the leading species in causing bacterial pneumonia which accounts for 31.7% [5]. Varying prevalence of bacterial pneumonia were reported in different parts of Ethiopia; 42.9% in southern Ethiopia and 32.1% in central Ethiopia, with *S. pneumoniae* and K. pneumoniae were predominant isolates, respectively [6, 7].

Bacterial pneumonia causes complications for everyone, but individuals with weakened immune systems, children, and the elderly are at higher risk [8]. Community and hospital-acquired pneumonia are the two main types of pneumonia. In the community, with a high prevalence, and it causes significant morbidity and mortality [9]. Patients living with HIV/AIDS especially those who had co-infection with one or more microorganisms, and older age individuals are more susceptible for infections with bacterial pneumonia [10]. The positive culture rate was slightly higher in women than in men and higher prevalence rates of lower respiratory tract infections were observed in age groups greater than or equal to 45 years [11].

The main problem concerning about treatment of bacteria causing pneumonia is multidrug resistance (MDR) (antibiotic resistance to at least three or more than three classes), extensively drug resistant (XDR) (resistance to all antibiotics classes except one), and pan-drug resistant (PDR) (resistance to all groups of antibiotics) [12, 13]. There are different mechanisms in which bacteria can escape from the effect of antibiotics. Resistance to one or more groups of antimicrobial agents may be innate or acquired by bacteria. The antibiotic resistance crisis is due to emerging and dissemination antibiotic resistance pathogen in the hospital and environments, inappropriate drug use, over use and consumption of drug resistant pathogens from animal sources and crops [14].

In China most frequently prescribed antibiotics including penicillin, erythromycin, tetracycline and clindamycin resistance were pertained by *S. aureus*, and *S. pneumoniae* was highly resistant to erythromycin, azithromycin and clindamycin. *E. coli*, was resistant to ampicillin, gentamicin, and ciprofloxacin. *K. pneumoniae*, has the highest resistance to gentamicin and ampicillin [15]. Similarly, cotrimoxazole was 100% resistant to *S. aureus* and *S. pneumoniae*. *K. pneumoniae* was resistant to most of the antibiotics showing more than 50% resistance to ceftriaxone and cefotaxime drugs respectively [16].

Nigeria’s Analysis of pneumonia-associated bacteria among HIV/AIDS patients in Nigeria showed that *P. aeruginosa* were highly resistant to all antibiotics including ciprofloxacin and ceftazidime whereas *E. coli*, *S. aureus* and *K. pneumoniae* were resistance to commonly prescribing drugs [17]. Gram-negative bacilli were highly resistant to ampicillin tetracycline, ciprofloxacin, and trimethoprim-sulfamethoxazole [7]. On the other hand, most of the isolates were less resistant to amikacin. Methicillin resistance was observed in isolates of *S. aureus* [18]. This study is the first systematic review and meta-analysis to report the national burden of bacterial pneumonia and MDR in Ethiopia; and it aimed to summarize the findings of local studies and estimate the pooled prevalence of bacterial pneumonia and MDR in Ethiopia.

## Methods

### Design and protocol registration

This systematic review and meta-analysis were designed to estimate the pooled prevalence of bacterial pneumonia and their multi-drug resistance pattern in Ethiopia based on the preferred reporting items for systematic review and meta-analysis protocols (PRISMA-P) [19]. The review protocol was registered in the international prospective register of systematic review (PROSPERO) under registration number CRD42023414098.

### Data source and search strategy

A comprehensive search of databases was performed to identify all relevant articles published on bacterial isolates with MDR of bacterial isolates from patients with pneumonia in Ethiopia from January 1, 2000 to April 2023. Articles published in English language were searched in PubMed, google scholar, scopus, science direct, African index medicos, African journal online (AJOL), Ethiopian journals, WHO afro library databases from April 6 to April 16, 2023. In addition to accounting for the studies’ omission during electronic database searches, a direct google search was carried out using listed references in included articles. The comprehensive and extensive searching strategy has been employed using condition, context, population, and outcome of interest (CoCoPop) formulating questions and searching terms were (‘‘prevalence”), (“epidemiology”) (“magnitude”), and (“bacterial pneumonia”) and (“antimicrobial resistance”), (“antibiotic resistance”) and (“antibiotic susceptibility”), (“hospital-acquired pneumonia”), (“community-acquired pneumonia”) and (“Ethiopia”). The search terms were combined using the Boolean operators “OR” and “AND” to fit the advanced searching of articles.

### Eligibility criteria

The authors developed a selection criteria checklist for study eligibility before identifying appropriately published, relevant full-text articles either in local or international journals. We included published and preprint (study done at Bahirdar University) of original articles that reported bacterial pneumonia and their antimicrobial resistance pattern in all age populations of Ethiopia, studies written in English, and laboratory-based observational (e.g., cross-sectional) studies. We excluded studies with no confirmation of bacterial isolates using phenotypic and/or genotypic methods, qualitative studies, review papers, commentaries, case series, case reports, conference proceedings, and abstracts.

### Data extraction

Data extraction was performed by four independent reviewers (HD, MT, OM, and HE) using a standard extraction format adapted from the Joanna Briggs Institute (JBI) data extraction format [20] and recorded them in a Microsoft Excel spreadsheet.

The extracted data includes, the first author’s name and year of publication, the study period, the study design, the study region, the total sample size, the number of isolates, the criteria for diagnosing bacterial isolates causing pneumonia, the number and percentage of Gram-positive and Gram-negative bacteria, and the prevalence MDR of commonly identified bacteria.

### Quality assessment

Four authors (MT, AG, HD, and MAB) carefully assessed the quality of the articles using JBI quality appraisal tool. The full texts of the articles were used to determine whether the study met the selection criteria or whether the eligibility of the article was called into question [30]. By using the critical appraisal checklists, studies with an average score of 50–75% were considered of good quality, while scores greater than 75% were considered of high quality. As a result, articles of both good and high quality were included for the analysis [31] (Supplementary Table 1).

### Outcome variables

Two findings were drawn from this systematic review and meta-analysis. The first goal was to determine the pooled estimates of bacterial pneumonia among pneumonia suspected Ethiopian patients. The second goal was to calculate the pooled prevalence of MDR of common pathogens.

### Data processing and analysis

The data were analyzed by using STATA version 14.0 statistical software. A random effect model was applied to estimate the pooled estimate and MDR of the isolates. A potential source of heterogeneity was investigated by subgroup and meta-regression analysis. The Cochran’s Q test and I^2^ statistics were used to quantify and assess the presence of heterogeneity between studies. The *p*-value of < 0.05 for I^2^ statistics was used to determine the presence of heterogeneity [21] and Der Simonian-Laired random effects model was employed [22]. Subgroup analysis was done based on the patient’s region, city, study design, and HIV sero-status. The results were presented using table and forest plot. Publication bias was evaluated using inspection of funnel plot symmetry and Egger’s test statistics. The Trim-and-Fill was used in asymmetrical funnel plots to integrate missing studies and estimate adjusted effect size. Meta-regression was also used to further assess the cause of heterogeneity.

## Result

### Selection and identification of studies

**Fig. 1:**
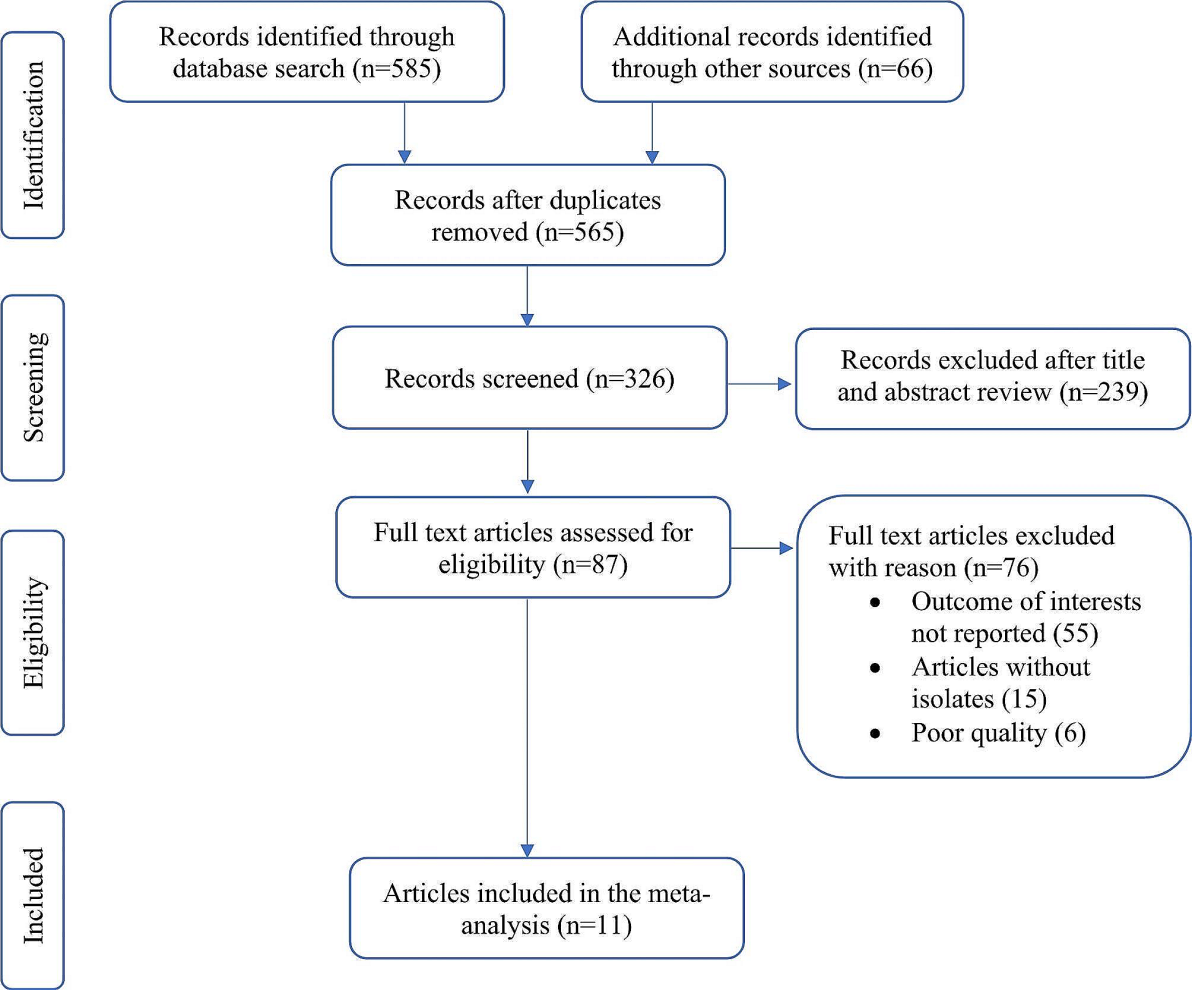
PRISMA flow diagram illustrating the process of selecting eligible studies for the systematic review and meta-analysis

A total of 651 articles were retrieved from databases. About 565 articles remained after removing 86 duplicate articles. From the remaining, 239 articles were excluded after reviewing the title, abstract, and objective of the study. Finally, 87 full-length articles were thoroughly reviewed by predetermined eligibility criteria, and 11 studies were included in the meta-analysis [18, 23–32] (Fig. 1).

### Characteristics of included studies

Table 1 Summarizes the characteristics of 11 studies included in our final meta-analysis. These studies were conducted between 2000 and 2023 in different regions of Ethiopia and were used to estimate the pooled prevalence and MDR of bacterial isolates suspected of pneumonia. From the total studies, 10 of the studies were cross-sectional and 1 study was prospective observational. All of the studies were published on peer review journals except one unpublished data that was obtained from preprint data [18, 23–32]. About 45.5% (5/11) of the studies were conducted in Amhara region [27–30, 32], followed by two studies 18.2% (2/11) in Addis Ababa city [23, 24], two in SNNPR [25, 26], one in Oromia region [31] and one in Tigray region [18]. The sample size of the studies included the least from Jimma 133 [31] to the highest from Addis Ababa 549 [23]. The reported prevalence of bacterial pneumonia ranged from 6.19% to in Addis Ababa [23] to 46.3% in Dessie [28], while the prevalence of MDR bacterial pneumonia ranged from 32.7% in Hawassa [26] to 84.6% in Dessie [28] (Table 1).

**Table 1:** Overview of included studies

Author	Study area	Region	Publication year	Study period	Study design	Study population	Sero status of HIV	Sample types (Culture specimens)	sample size	Total pathogen isolated	Prevalence %	MDR
Negash et al. [23]	Addis Ababa	Central	2019	September 2016 to August 2017	prospective observational	Children	negative	Blood	549	34	6.19	-
Nurahmed et al. [24]	Addis Ababa	Central	2020	May–July 2018	Cross-sectional	> 18 years old	negative	Sputum	240	72	32.1	80.3
Regasa et al [25]	Arba Minch	SNNPR	2014	February to May 2013	Cross-sectional	Adult	negative	Sputum	170	73	42.9	60.3
Gebre et al [26]	Hawassa	SNNPR	2021	July to October 2019	Cross-sectional	> 18 years old	negative	Sputum	406	138	33.5	32.4
Temesgen et al [27]	Bahir Dar	Amhara	2019	April to July 2018	Cross-sectional	Adult	negative	Sputum	414	167	40.3	76
Tilahun al [28]	Dessie	Amhara	2023	January to April 2021	Cross-sectional	all age group	positive	Sputum	378	146	46.3	84.6
Dessie et al [29]	Dessie	Amhara	2021	February to April 2020	Cross-sectional	all age group	both	Sputum	406	157	38.7	63.1
Assefa et al [30]	Gondar	Amhara	2022	April to June 2021	Cross-sectional	Adult	negative	Sputum	312	126	39.4	72.2
Regasa et al [31]	Jimma	Oromia	2015	March to July 2012	Cross-sectional	> 18 years old	negative	Sputum and blood	133	60	45	62.7
Adhanom et al [18]	Mekelle	Tigray	2019	August-December 2016	Cross-sectional	> 18 years old	positive	Sputum	252	110	43.7	67.9
Genetu et al [32]	Bahir Dar	Amhara	2023	February to June 2019	Cross-sectional	> 18 years old	positive	Sputum	163	68	41.7	77.9

### The pooled prevalence of bacterial pneumonia

**Fig. 2:**
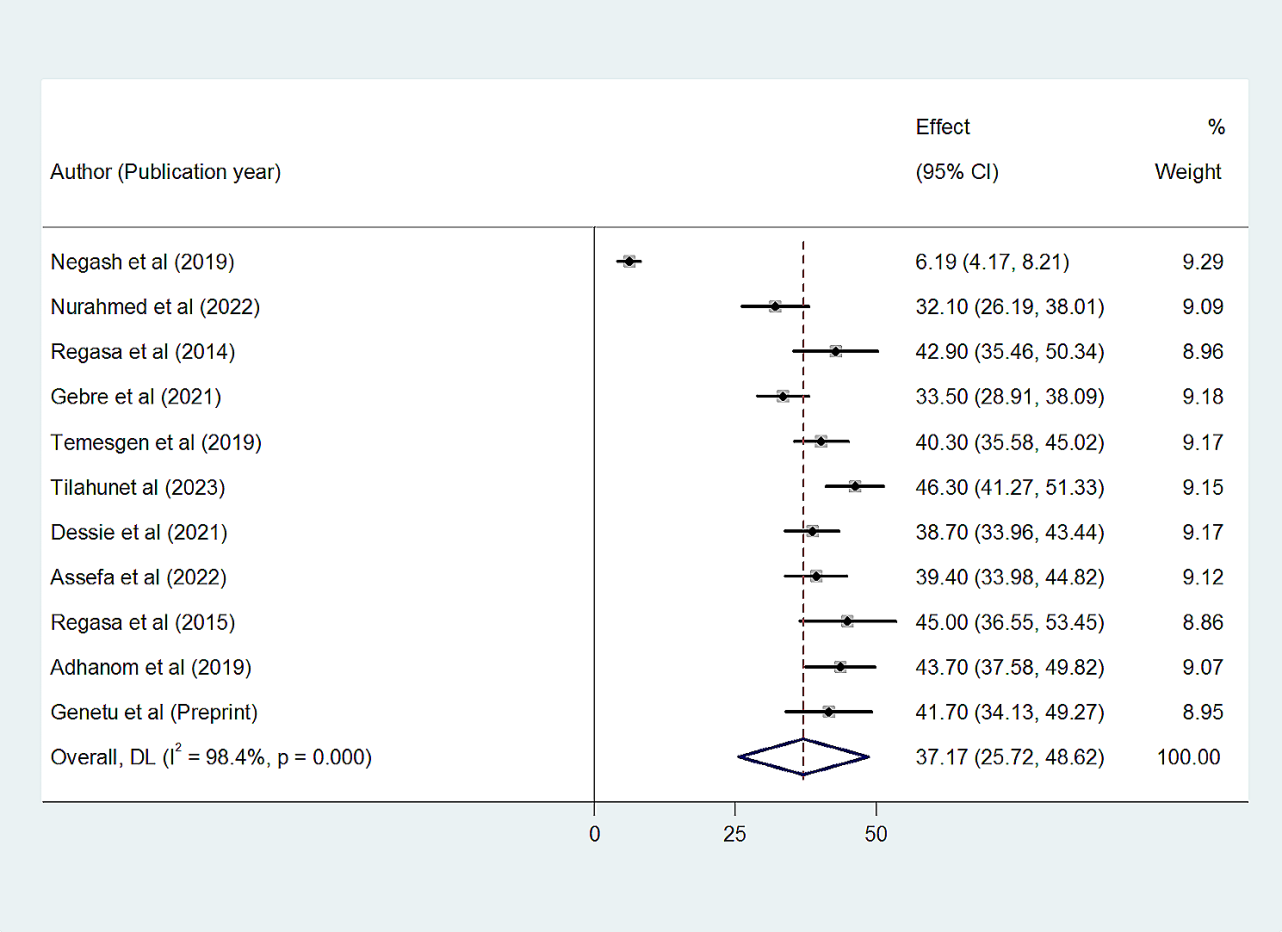
Forest plot showing the overall prevalence of bacterial pneumonia in Ethiopia

A total of 11 studies reported that bacterial pneumonia infections were detected in 1151 samples out of a total of 3423 samples taken from bacterial pneumonia suspected patients who visit the health care system. In this systematic review and meta-analysis, the overall pooled prevalence of bacterial pneumonia in Ethiopia was 37.17% (95% CI 25.72–48.62%) with substantial heterogeneity (I^2^ = 98.4%, *p* < 0.001) across the studies. (Fig. 2).

### Subgroup analysis

Subgroup analyses revealed 38.71% (95% CI: 33.37–44.05, I^2^ = 72%, *p* < 0.001) and 42.45% (95% CI 35.00–49.90, I^2^ = 78.5%, *p* = 0.031) pooled prevalence of bacterial pneumonia in the age group > 18 years and studies involving all age groups, respectively, with no statistically significant difference (*p* = 0.14). Another subgroup analysis was performed on HIV sero-status and indicated that the prevalence of bacterial pneumonia among HIV-negative patients was 34.07% (95% CI 19.32–48.83, I^*2*^ *=* 98.6%, *p* < 0.001) while it was 44.51% (95% CI 44.06–47.97, I^*2*^ *=* 0%, *p* = 0.582) in HIV positive patients. Lastly, subgroup by study area showed that the prevalence of bacterial pneumonia was 18.99% in Addis Ababa city, 37.73% SNNP region, and 41.21% in Amhara region with no significant difference across the regions (*p* = 0.46). The prevalence of bacterial pneumonia pooled from studies showed increment from the period ≤ 2020 (35.49%, 95% CI: 14.51, 56.48) to > 2020 with prevalence of 38.55% (95% CI: 34.23, 42.86). On the other hand, the prevalence of bacterial pneumonia in terms of sample size ≤ 384 was 41.45% (95% CI: 37.62, 45.29) (Table 2).

**Table 2:** Subgroup analysis of bacterial pneumonia by region, HIV sero-status, publication year and sample size

Subgroups	Category	No of studies	No of isolates tested, N	Pooled prevalence of N (%)	95% CI	Heterogeneity test (I^2^)	*P*-value	Heterogeneity between groups (*p*-value)
Population	Children	1	34	6.19	(4.17, 8.21)	< 0%	< 0.001	< 0.001
	> 18 years	5	448	38.71	(33.37, 44.05)	72.0%	0.006	
	Adult	3	366	40.47	(37.26, 43.08)	< 0%	0.754	
	All age group	2	303	42.45	(35.00, 49.90)	78.5	0.031	
	Total pooled	11	1151	37.17	(25.72–48.62)	98.4%%	< 0.001	
Region	Central/ Addis Ababa	2	106	18.99	(6.40, 44.38)	98.5%	< 0.001	0.032
	Amhara	5	664	(41.21)	(38.39, 44.03)	28.61%	0.231	
	Oromia	1	60	(45.00)	(36.55, 53.45)	0.0%	< 0.001	
	Tigray	1	110	(43.70)	(37.58, 49.82)	0.0%	< 0.001	
	Hawassa/SNNPR	2	211	(37.73)	(28.56, 46.89)	77.5%	0.035	
	Total pooled	27	1151	37.17	(25.72–48.62)	98.4%%	< 0.001	
Publication year	≤ 2020	5	444	(35.49)	(14.51, 56.48)	98.9%	< 0.001	0.780
	> 2020	6	707	(38.55)	(34.23, 42.86)	73.8%	0.002	
	Total pooled	11	1151	37.17	(25.72–48.62)	98.4%%	< 0.001	
Sero-status	HIV-positive	3	324	44.51	(41.06, 47.97)	0.0%	0.582	0.083
	HIV-negative	7	670	34.07	(19.32, 48.83)	98.6%	< 0.001	
	Both	1	157	38.70	(33.96, 43.44)	0.0%	< 0.001	
	Total pooled	11	1151	37.17	(25.72–48.62)	98.4%%	< 0.001	
Sample size	≤ 384	7	657	(41.45)	(37.62, 45.29)	60.03%	0.002	0.255
	> 384	4	494	(29.61)	(9.57, 49.64)	99.1%	< 0.001	
	Total pooled	11	2348	315 (14.52)	(11.59, 17.44)	93.0%	< 0.001	

### The pooled prevalence of MDR

**Fig. 3:**
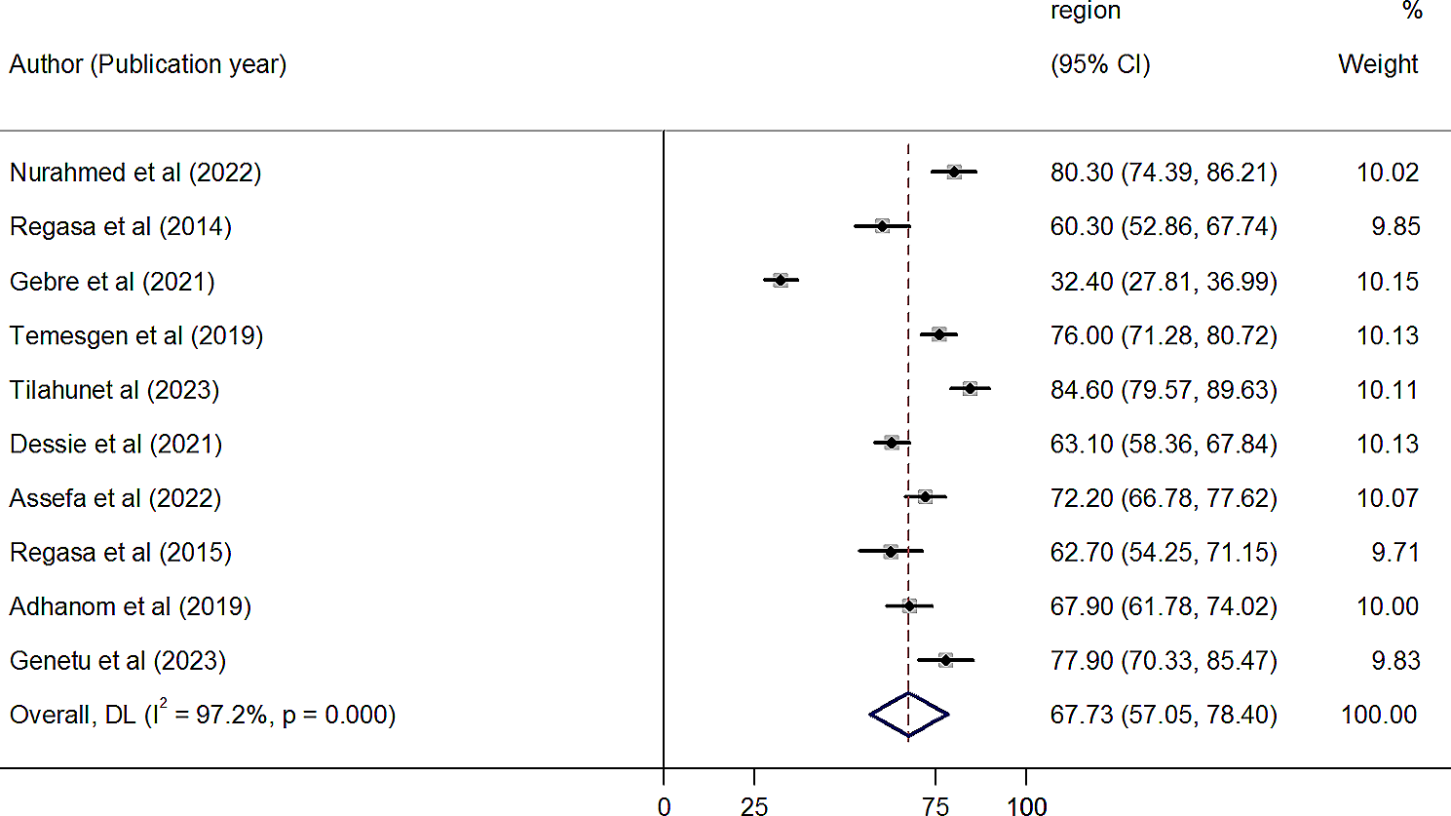
Forest plot showing overall pooled prevalence of MDR isolates causing bacterial pneumonia in Ethiopia

**Fig. 4:**
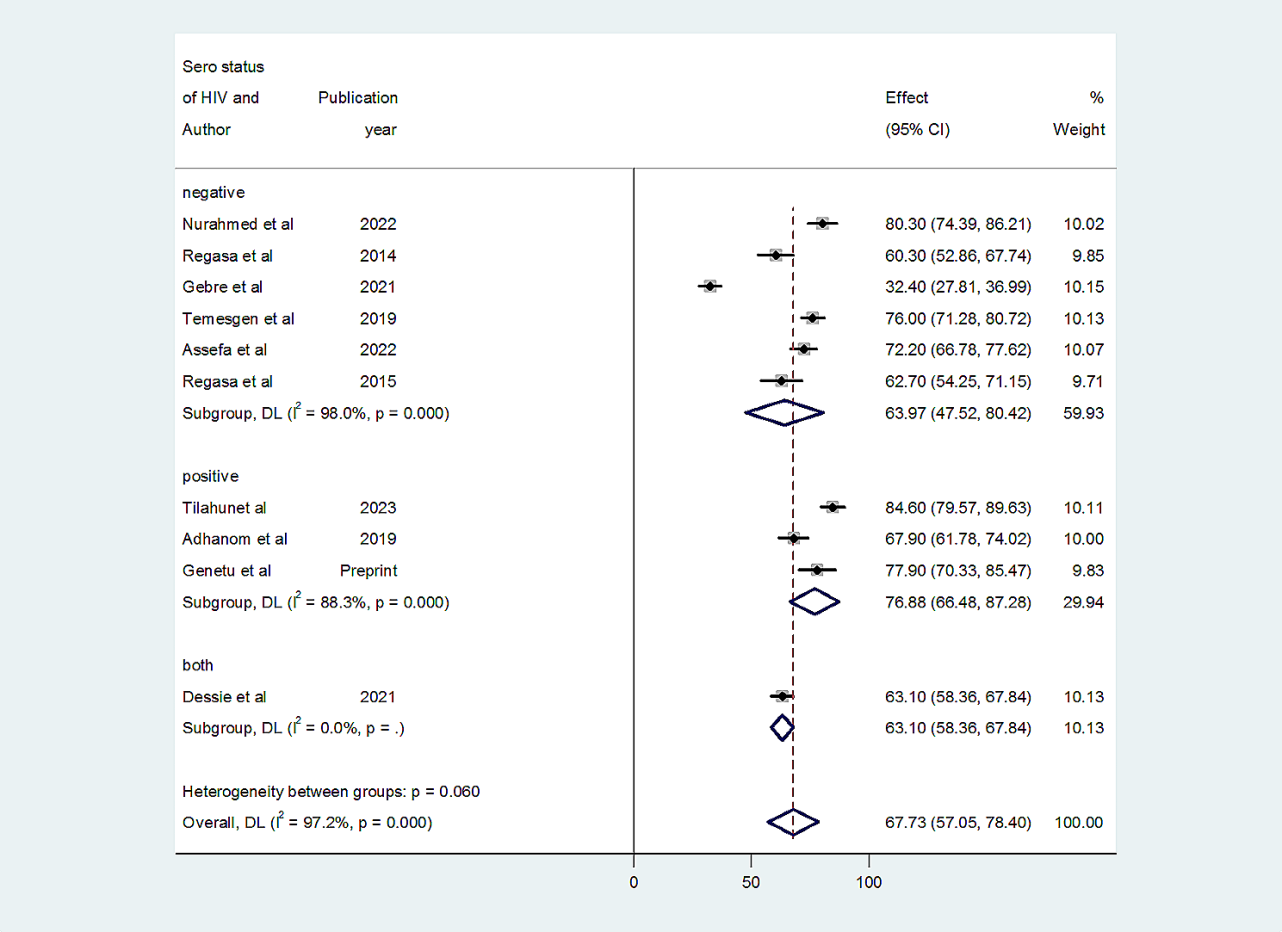
Sub group analysis of MDR bacterial isolates by HIV sero-status bacterial pneumonia suspected patients in Ethiopia

In individual study, the magnitude of MDR in Ethiopia was varying from 32.4 to 84.6%. The overall pooled prevalence of MDR of bacteria isolated from patients with pneumonia in Ethiopia was 67.73% (95% CI: 57.05–78.40) with high level of heterogeneity (I^2^ = 97.2%, *p* < 0.001) across the studies (Fig. 3). Subgroup analysis performed on HIV serostatus indicated that the prevalence of MDR among HIV positive patients was 76.88% (95% CI: 66.48–87.28%, I^*2*^ *=* 88.3%, *p* < 0.001) while it was 63.97% (95% CI: 47.52–80.42%, I^*2*^ *=* 98.0%, *p* < 0.001) in HIV negative patients (Fig. 4).

### Pooled prevalence of pneumonia causing bacterial isolates

Summary of Table 3 indicated eleven different types of bacterial isolates. Gram-negative bacteria were the predominant isolates with 61.5% pooled prevalence. The most common bacterial isolate was *K. pneumoniae* with an overall prevalence of 21.97% (95% CI 16.11–27.83%), followed by *S. pneumoniae* 17.02 (95% CI 9.19–24.86%), *S.aureus* 14.47% (95% CI 8.95–19.99%), *P. aeruginosa* 9.98% (95% CI 6.57–14.13%), *E. coli* 9.75% (95% CI 7.01–12.50), *Citrobacter species* 4.12% (95% CI 1.84–6.39%), *Enterobacter species* 5.10% (95% CI 0.57–9.63%), *H. influenzae* 3.89%(95% CI 2.45–5.32%), *P. mirabilis* 2.73% (95% CI 1.20–4.27%), *P. vulgaris* 1.71% 95% CI -0.92–4.33%) and *Acinetobacter species* 3% 4.70% (95% CI -0.87–8.54% ) (Table 3).

**Table 3:** Bacterial isolates from pneumonia-suspected patients in Ethiopia

Bacteria	Number of studies	No. of bacteria isolates	Pooled Prevalence (95% CI)	I^2^ (%)	*p*-value
*S. pneumoniae*	11	265	17.02 (9.19–24.86)	96.5	< 0.001
*S. aureus*	11	179	14.47(8.95–19.99)	92.9	< 0.001
*K. pneumoniae*	11	290	21.97(16.11–27.83)	93.7	< 0.001
*E. coli*	11	142	9.75(7.01–13.39)	80.6	< 0.001
*P. aeruginosa*	11	113	9.98 (6.57–14.13)	74.6	< 0.001
*Citrobacter species*	4	26	4.12(1.84–6.39)	30.2	0.22
*Enterobacter species*	6	25	5.10(0.57–9.63)	82.7	< 0.001
*H. influenzae*	9	49	3.89(2.45–5.32)	0	0.499
*Proteus mirabilis*	7	22	2.73(1.20–4.27)	0	0.994
*Proteus vulugaris*	5	10	1.71(-0.92-4.33)	0	0.986
*Acinetobacter species*	6	32	4.70(0.87–8.54)	77	< 0.001

### Sensitivity analysis

According to our sensitivity analysis finding, each study did not affect the pooled estimate of the proportion indicating the precise aggregate result. When individual studies were omitted, the pooled effect size lay within the 95% confidence interval of the overall pooled effect size. This demonstrated that no single study had an impact on the overall pooled prevalence of bacterial pneumonia infection in Ethiopia (Table 4).

**Table 4:** Sensitivity analysis of the included studies

Author	Estimate	95% CI
Negash et al. [23]	40.1	37.1–43.1
Nurahmed et al. [24]	37.7	25.2-50.12
Regasa et al [25]	36.6	24.5–48.7
Gebre et al [26]	37.5	24.8–50.2
Temesgen et al [27]	36.9	24.-5-49.2
Tilahun et al [28]	36.2	24.3–48.3
Dessie et al [29]	37.01	24.6–49.5
Assefa et al [30]	36.9	24.6–49.3
Regasa et al [31]	36.4	24.4-48.43
Adhanom et al [18]	36.5	24.42–48.6
Genetu et al [32]	36.7	24.6–48.6
**Combined**	**37.17**	**25.71–48.62**

### Publication bias

**Fig. 5:**
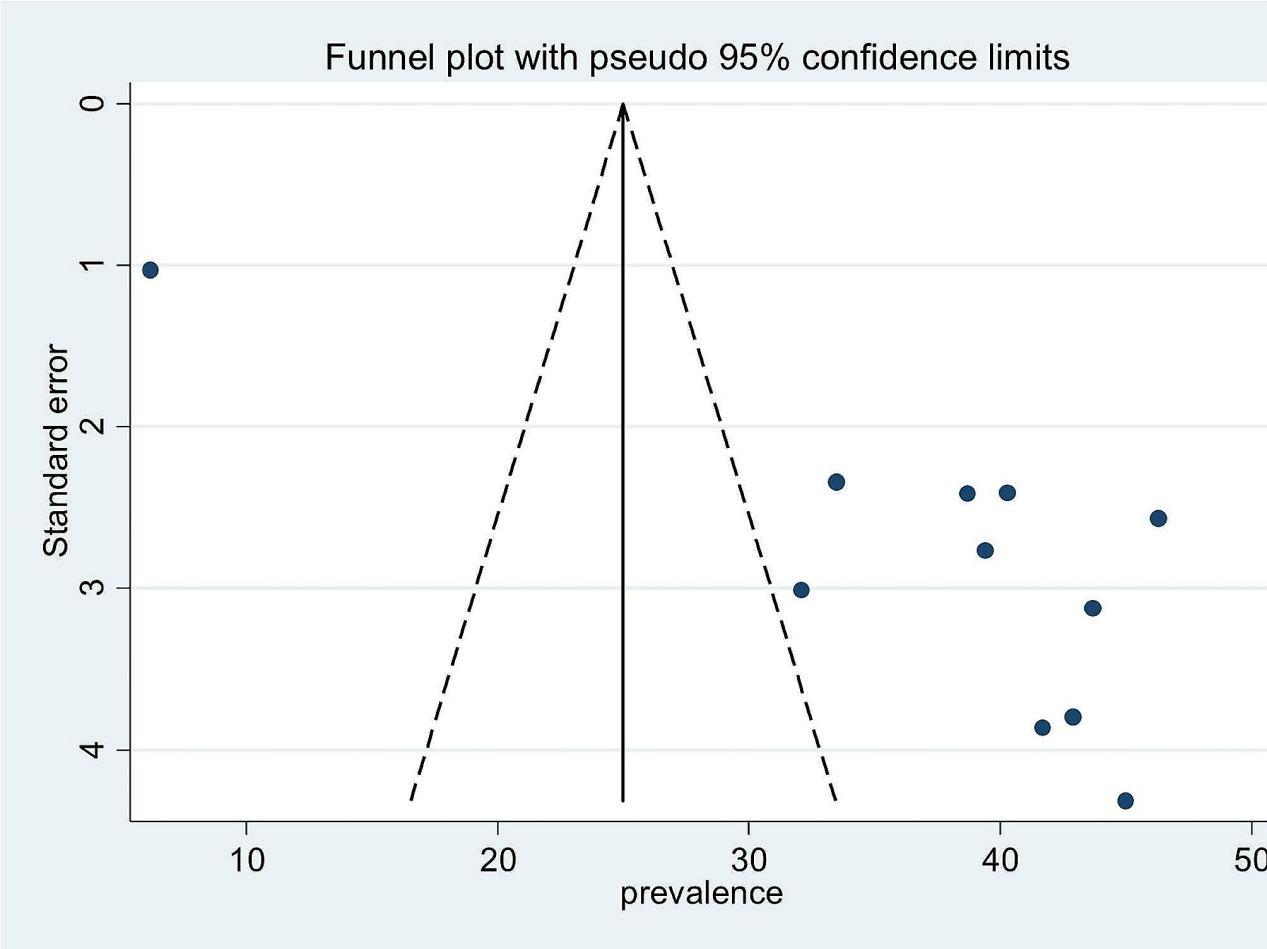
Funnel plot on the prevalence of bacterial pneumonia in Ethiopia illustrating the presence of publication bias

**Fig. 6:**
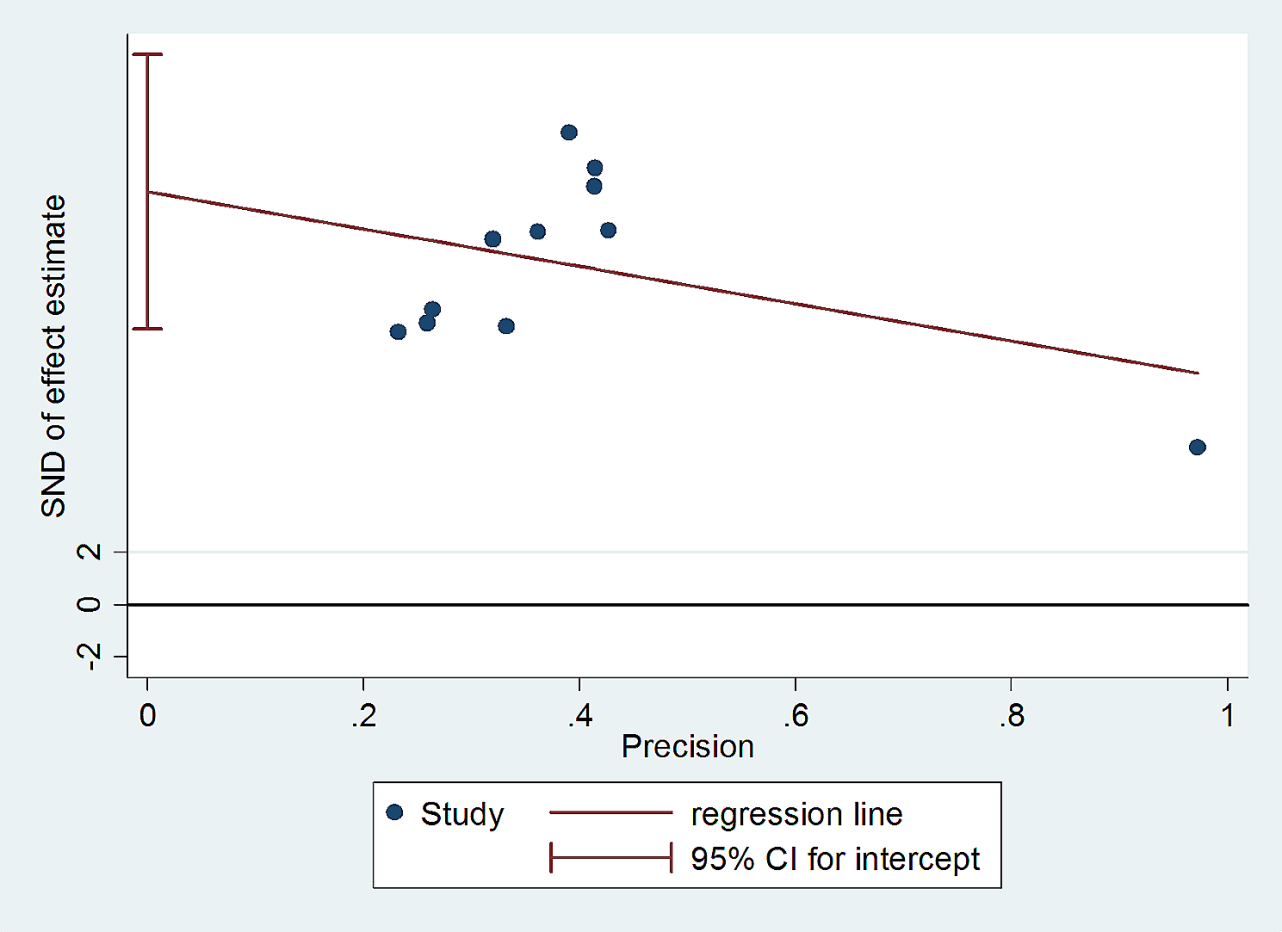
Egger’s test graph depicting publication bias

The funnel plot was used to assess the impact of the small-studies effect or publication bias on estimated pooled prevalence. In this study, the asymmetry of the funnel plot illustrated the presence of publication bias with over 63.6% of the studies skewed to the right side of the triangular zone (Fig. 5). Furthermore, Egger’s test statistics also confirmed the presence of significant publication bias at a *P*-value < 0.001 (Table 5) (Fig. 6).

**Table 5:** Egger’s test statistics of the prevalence of bacterial pneumonia in Ethiopia

Std-Eff	Coef.	Std. Err.	t	*P*	95% CI
Slope	-7.31	5.23	-1.36	0.206	-18.95, 4.7
Bias	15.78	2.31	6.82	< 0.001	10.55, 20.03

### Trim and fill analysis of the pooled prevalence of bacterial pneumonia in Ethiopia

Due to the presence of slightly significant publication bias, we performed a trim and fill analysis. After incorporating 6 studies, the trim and fill analysis revealed a pooled prevalence of bacterial pneumonia in Ethiopia was 21.33% (95% CI:10.86–31.798) (Table 6).

**Table 6:** Trim and fill analysis of the prevalence of bacterial pneumonia in Ethiopia

Method	Pooled est.	95% CI		Asymptotic		No. of studies
		Lower	Upper	z-value	*p*-value	
Fixed	24,998	23.664	26.333	36.709	< 0.001	11
Random	37.167	25.716	48.617	6.362	< 0.001	
Test for heterogeneity: Q = 619.951 on 10degrees of freedom (*p* < 0.001)						
Moment-based estimate of between studies variance = 366.490						
Trimming estimator: Linear						
Meta-analysis type: Fixed-effects model						
Iteration	**Estimate**	**Tn**	**# To trim**		**Diff**	
1	24.998	57	5		66	
2	19.979	63	6		12	
3	17.708	65	6		4	
4	17.708	65	6		0	
Filled						
Meta-analysis						
Method	**Pooled est.**	**95% CI**		**Asymptotic**		**No. of studies**
		**Lower**	**Upper**	**z-value**	***p***-**value**	
Fixed	17.708	16.531	18.885	29.489	< 0.001	17
Random	21.331	10.863	31.798	3.994	< 0.001	
Test for heterogeneity: Q = 1138.521 on 10 degrees of freedom (*p* < 0.001)						
Moment-based estimate of between studies variance = 474.990						

### Meta-regression

Meta-regression was carried out to further explore the potential sources of heterogeneity or variability among studies included in the meta-analysis. We included continuous study characteristics as covariates including published year, sample size, and number of isolates. In this study, total isolates and sample size were the responsible variables for the existence of heterogeneity between studies (*P* < 0.001) (Table 7).

**Table 7:** Meta-regression analysis of prevalence and MDR pattern of bacterial pneumonia by different categories of studies included in the systematic review and meta-analysis

	Type of variables	Exp(b)	SE	t	*P*	95% CI
Prevalence	Total isolates	1.212	0.031	7.60	< 0.001*	1.141801–1.287055
	Publication year	0.858	0.418	-0.31	0.762	0.2715376 − 2.711604
	Sample size	0.928	0.0089	-8.58	< 0.001*	0.9088477 0.9471566
MDR	Total isolates	2.283	1.020	1.85	0.114	0.7652871 − 6.812579
	Publication year	26.789	43.822	2.01	0.091	0.4893512 − 1466.579
	Sample size	0.701	0.119	-2.10	**0.081**	0.4636661 − 1.061176

*= significant causes of heterogeneity

## Discussions

Bacterial pneumonia is one of the most serious public health issues due to the high medical and economic costs that result in increased morbidity and mortality in people of all ages worldwide [33]. Bacterial pneumonia is characterized by a productive cough, fever with shaking chills, shortness of breath, sharp chest pain during deep breaths, increasing rate of breathing and confusion may be the most noticeable symptom in the elderly [34]. The main aim of this study was to determine the pooled prevalence of bacterial pneumonia and MDR of bacterial isolates causing pneumonia in Ethiopia, involving about 3428 study participants.

The overall pooled prevalence of bacterial pneumonia in Ethiopia was found to be, 37.17% (95% CI 25.72–48.62%) with a high level of heterogeneity (I^2^ = 98.4%, *p* < 0.001). This finding is comparable with a previous review reporting the pooled prevalence of bacterial pneumonia in Sudan (33.33%), and a systematic review and meta-analysis of pneumonia in east African children (34%) [35], Sudan (42%) [36], Asian countries (44.8%) [37], Iran (44%) [38] and India (46.3%) [39]. On the contrary, the finding of the present systematic review and meta-analysis is massively higher than systematic review and meta-analysis on the pooled magnitude of pneumonia among under-five children in Ethiopia which accounted 18.03 [40] and lower than the study in Ghana 84.5% [41], Nigeria 69.6% and 45.2% [42, 43], Zambia 59% [44], Egypt 50.4% [45], Pakistan 75% (37), in different regions of India 52.83% and 58.8%, 83% [46, 47], Bangladesh 61.83% [48], multicenter study in China 74.4% [49], Spain 50.7% [50], and Vietnam 61.8% [51]. This could be due to differences in the study setting, genetic background of the study population, and sample size. Another reason for this discrepancy could be methodological differences as some studies use molecular and serological detection methods for both typical and atypical pneumonia [52].

In this meta-analysis, Gram-negative bacteria accounted for 61.5% of culture-positive samples. Similarly, another review article reported 76.13 to 95.3% of Gram-negative bacteria as the cause of bacterial pneumonia infections [53]. The high prevalence of Gram-negative bacteria in various research is due to differences in sample size, geographic location, study period, study population, and respiratory flora specimen contamination.

Regarding the specific bacteria identified, the most common bacterial isolate causing pneumonia was *K. pneumoniae* with an overall prevalence of 21.97% (95% CI 16.11–27.83), followed by S. pneumoniae 17.02 (95% CI 9.19–24.86). Similar findings from Cambodia reported *K. pneumoniae* as the leading cause [54]. According to a study in Nepal, the most common bacterial isolate were *K. pneumoniae* (27.0%), *S. aureus* 20.8%, *S. pneumoniae 18*.8%, *E. coli* 8.3%, *H. influenzae, K. oxytoca*, *P. aeruginosa*, 4.2% each, *Enterobacter spp* 2.1% and unidentified Gram negative bacteria 10.4% [11]. In Nigeria, *K. pneumoniae* (23%) was the predominant, followed by *S. aureus* (21%), *S. pneumoniae* (13%), *P. aeruginosa* (9%) and *E. coli* (3%) [55]. Another comparative cross-sectional also indicated, *K. pneumoniae* was the predominant bacteria isolated 16 (13.3%) followed by *E. Coli* 10 (8.3%), *S. pneumoniae* 10 (8.3%), *S. aureus* 9(7.5% ), *P. aeruginosa* 5 (4.1% ), *M. catarrhalis* 4 (3.3% ) and *H. influenzae* 2 (1.6%) [17]. Different reported *S. pneumoniae* was the most frequent bacteria isolated from the sputum culture of community-acquired pneumonia and *K. pneumoniae* were the second frequent pneumonia causing bacteria [56]. A similar finding was reported from a community-based study depicting *S. pneumoniae, H. influenzae*, *S. aureus* as predominantly isolated bacterial [3]. In contrast in the UK, *S. pneumoniae* was the most commonly isolated species (30%) followed by *H. influenzae* (19%) and *M. catarrhalis* (2%) [57]. Another descriptive cross-sectional study was conducted in Malawi, the predominant isolate were *S. aureus* followed by *P. aeruginosa*, *E. cloacae*, and *K. pneumonia* [58].

Furthermore, the pooled prevalence of multi-drug resistant bacterial pneumonia isolates was 67.73% (95% CI: 57.05–78.40). This finding was in line with studies conducted in Nigeria (67.2%) [59] and systematic review report of 59.7% overall MDR prevalence in Ethiopia [60], and lower than the study conducted in Cameron 79.4% [61]. This finding also alarms the need for integrated efforts of antimicrobial surveillance systems and poses for the development of new antibiotics.

In the current review, substantial heterogeneity with an (I^2^ = 97.2%, *p* < 0.001) was found. This study’s substantial heterogeneity is most likely not attributable to publication bias, but rather to variances in methodological concerns such as sample size, target population categories, and patient underpinning circumstances. The other difference could be attributed to the target group from which samples were collected and the antibiotic resistance crisis, primarily because antibiotics lose their efficacy over time due to the emergence and spread of resistance among bacterial pathogens, which is primarily caused by the overuse and inappropriate use of antibiotics, as well as the widespread use of antibiotics in agriculture and the food industry. Antibiotic resistance is a natural phenomenon in bacteria that cannot be stopped; however, various measures can be taken to reduce the rate of its development and devise more effective strategies to control its spread.

Sensitivity analysis, sub-group analysis, and meta-regression have been carried-out to rule out the most possible causes of heterogeneity. The results of sensitivity analysis proved that there is no single study that impacted the pooled effect size. The pooled prevalence of bacterial pneumonia infections in Ethiopia was calculated by omitting each study sequentially and the computed pooled prevalence was within 95% CI of the overall pooled prevalence. Meta-regression has confirmed that a number of total pathogens isolates and sample size were a significant cause of heterogeneity in prevalence of bacterial pneumonia while publication year was not found to be a significant cause. In addition to this, publication bias was assessed using funnel plot and Egger’s test statistics, and trim and fill analysis was performed to fill the bias.

One of the notable strengths of this study is its comprehensive nature, being the first of its kind to conduct a thorough analysis of bacterial pneumonia and MDR within Ethiopia. It encompasses a wide range of studies conducted across multiple regions and cities of the country, providing a robust overview.

Furthermore, the study included various studies done in different target populations using clinical specimens to show a clear picture of bacterial pneumonia and MDR in the country. However, the results should be interpreted with caution, as the reviewed studies were highly heterogeneous in terms of prevalence, aetiology, study setups, study participants, disease conditions, clinical specimens, sample sizes, and AST methods. Therefore, to account for this heterogeneity, the random-effects model of Der Simonian and Laird was implemented in the meta-analyses. Moreover, subgroup analyses, sensitivity analysis, and meta-regression were conducted to further address and mitigate the impact of heterogeneity on the findings.

## Conclusion

According to this systematic review and meta-analysis, the pooled prevalence of bacterial pneumonia infection and MDR have alarmingly increased and become a public health threat. The most common etiology identified was *K. pneumoniae* followed by *S. pneumoniae*. This indicates an urgent need of routine screening and appropriate treatment for better management of pneumonia suspected patients as well as effective controlling of the emergence of drug resistance. Furthermore, it serves as a wake-up call to international, continental, and national health bureaus, as well as other stakeholders, to develop targeted prevention and control strategies, and strengthen antibiotics stewardship programs for better management of hospital-acquired as well as community-acquired infections. Moreover, the data could be used for future complementary research and evidence-based decision-making both in clinical and public health approaches.

### Electronic supplementary material

Below is the link to the electronic supplementary material.

Supplementary Material 1

Supplementary Material 2

## Data Availability

All relevant data are included in the manuscript and its supplementary data.

## Abbreviations

AMR: Antimicrobial resistance
CI: Confidence interval
CLSI: Clinical Laboratory Standards Institute
MDR: Multidrug resistance
PRISMA: Preferred Reporting Items for Systematic Reviews and Meta-Analyses
STATA: statistics and data
WHO: World Health Organization
